# Advancements in the understanding and management of histiocytic neoplasms

**DOI:** 10.1007/s44313-024-00022-w

**Published:** 2024-07-04

**Authors:** Kyung-Nam Koh, Su Hyun Yoon, Sung Han Kang, Hyery Kim, Ho Joon Im

**Affiliations:** Division of Pediatric Hematology/Oncology, Department of Pediatrics, Asan Medical Center Children’s Hospital, University of Ulsan College of Medicine, 88, Olympic-ro, 43-gil, Songpa-gu, Seoul, 05505 Republic of Korea

**Keywords:** Histiocytic, Neoplasms, Langerhans cell histiocytosis, Erdheim-Chester disease, Rosai-Dorfman disease, Juvenile xanthogranuloma, Genetic mutation

## Abstract

Histiocytic neoplasms are rare diseases involving macrophages, dendritic cells, and monocytes. They include Langerhans cell histiocytosis (LCH), Erdheim-Chester disease (ECD), Rosai-Dorfman disease (RDD), juvenile xanthogranuloma (JXG), and histiocytic sarcoma. Histiocytic neoplasms are characterized by varied clinical courses and prognoses, necessitating a nuanced understanding of their classification, epidemiology, and clinical manifestations. Genetic studies have revealed somatic mutations, predominantly in the MAPK pathway, suggesting a clonal neoplastic nature. This review covers the current understanding of histiocytic neoplasms, molecular pathophysiology, with a particular focus on mutations in genes such as BRAF, MAP2K1, and the PI3K-AKT signaling pathways, and evolving treatment strategies, especially focusing on LCH, ECD, RDD, and JXG. The treatment landscape has evolved with advancements in targeted therapies. BRAF inhibitors, such as vemurafenib and dabrafenib, have shown efficacy, especially in high-risk LCH cases; however, challenges remain, including relapse post-treatment discontinuation, and adverse effects. MEK inhibitors have also demonstrated effectiveness, and cobimetinib has recently been approved for use in adults. Further research is required to determine the optimal treatment duration and strategies for managing therapy interruptions. Advancements in molecular genetics and targeted therapies have revolutionized the management of histiocytic neoplasms. However, ongoing research is crucial for optimizing patient outcomes.

## Introduction

Histiocytic neoplasms encompass a range of rare diseases characterized by the proliferation of macrophages, dendritic cells, and monocyte-derived cells in various tissues and organs [1]. These disorders include Langerhans cell histiocytosis (LCH), Erdheim-Chester disease (ECD), Rosai-Dorfman disease (RDD), juvenile xanthogranulomas (JXG), histiocytic sarcomas (HS), and indeterminate cellular histiocytosis (ICH). These neoplasms, also referred to as histiocytosis or histiocytic/dendritic cell neoplasms, fall under the broader category of hematologic disorders. They are notable for the accumulation of myeloid dendritic cell-derived neoplastic cells accompanied by inflammatory infiltrate [2, 3].

The terms ‘histiocytosis’, ‘histiocytic disorders’, ‘histiocytic neoplasms’, and ‘histiocytic/dendritic cell neoplasms’ are used interchangeably. The term ‘histiocyte’ is used to morphologically describe tissue-resident macrophages [1]. Dendritic cells, monocytes, and macrophages play integral roles in the mononuclear phagocyte system. Diseases resulting from the abnormal expansion and accumulation of histiocytes and dendritic cells are collectively known as ‘histiocytosis’. Histiocytosis refers to a broad spectrum of diseases, including neoplastic diseases, which arise from the clonal expansion of histiocytic/dendritic cells, and reactive immune-dysregulated diseases, such as haemophagocytic lymphohistiocytosis, caused by overactivated macrophages. This review focuses primarily on clonal neoplastic diseases that affect histiocytic/dendritic cells.

Recurrent genetic alterations have been identified in various histiocytic neoplasms. These alterations predominantly consist of somatic missense mutations, indels, and fusions in genes related to the mitogen-activated protein kinase (MAPK) signaling pathway [3–6]. As a result, many histiocytic disorders are now recognized as clonal neoplastic diseases characterized by the constitutive activation of the MAPK pathway.

This review aims to provide an overview of the molecular pathophysiology, focusing on mutations in genes such as those involved in the MAPK and PI3K-AKT signaling pathways and discusses the evolving treatment strategies for histiocytic neoplasms, focusing on conditions, with a particular focus on the four major types: LCH, ECD, RDD, and JXG.

## Classification

Histiocytic neoplasms represent a heterogeneous class of diseases with varied clinical courses and prognoses, including LCH, ECD, RDD, JXG, HS, and ICH [7]. The classification of histiocytic neoplasms has evolved over time, reflecting the advancements in our understanding of these disorders. Initially, the Working Group categorized histiocytoses in 1987 into three primary groups: Langerhans, non-Langerhans, and malignant histiocytoses [8]. However, this classification has faced challenges as emerging deep sequencing diagnostic methods have revealed shared molecular features between neoplasms classified as LCH and ECD [1–3]. In response to these insights, the Histiocyte Society revised its classification in 2016 [1]. This new framework incorporates clinical, radiographic, histological, phenotypic, and molecular features, dividing the disease into five groups: (1) Langerhans-related histiocytosis; (2) cutaneous and mucocutaneous histiocytosis; (3) malignant histiocytosis; (4) Rosai-Dorfman disease; and (5) hemophagocytic lymphohistiocytosis and macrophage activation syndrome.

Further refinement is seen in the 5th edition of the WHO classification, which places dendritic cells and histiocytic neoplasms after myeloid neoplasms, recognizing their origin from common myeloid progenitors [7] (Table 1). This classification organizes neoplasms into three main groups: 1) plasmacytoid dendritic cell neoplasms, 2) Langerhans cells and other dendritic cell neoplasms, and 3) histiocytic neoplasms. The plasmacytoid group included mature forms associated with myeloid neoplasms and blastic variants. Langerhans cell neoplasms cover LCH and sarcomas. Other dendritic cell neoplasms include indeterminate and interdigitated cell tumors. Histiocytic neoplasms encompass a spectrum of disorders including JXG, ECD, RDD, ALK-positive histiocytosis, and histiocytic sarcoma, each with distinct clinical implications [7].

**Table 1:** Classification of dendritic cell and histiocytic neoplasms in the 5th edition of the World Health Organization classification of hematolymphoid tumors

**Dendritic cell and histiocytic neoplasms**
1. Plasmacytoid dendritic cell neoplasms
• Mature plasmacytoid dendritic cell proliferation associated with myeloid neoplasm
• Blastic plasmacytoid dendritic cell neoplasm
2. Langerhans cell and other dendritic cell neoplasms
1) Langerhans cells neoplasms
• Langerhans cell histiocytosis
• Langerhans cell sarcoma
2) Other dendritic cell neoplasms
• Indeterminate dendritic cell tumor
• Interdigitating dendritic cell sarcoma
3. Histiocytic neoplasms
• Juvenile xanthogranuloma
• Erdheim-Chester disease
• Rosai-Dorfman disease
• ALK-positive histiocytosis
• Histiocytic sarcoma

## Epidemiology and clinical presentation

The epidemiology and clinical presentation of histiocytic neoplasms reveal significant variations in the age of onset, sex predilection, racial/ethnic distribution, and affected organ systems, underlining their distinct pathophysiological origins.

LCH predominantly affects young children, with the highest incidence observed between 0–4 years of age along with male predominance. The incidence decreases with age, with rates of 5–9 per million in children under 15 years of age and approximately 1 per million in older patients [2]. LCH typically manifests as bone involvement and often presents as a unifocal bone disease. Although LCH commonly affects the bones and skin, it can also affect any organ, including the liver, spleen, lungs, lymph nodes, central nervous system (CNS), and hematopoietic system(4).

ECD is mainly diagnosed in older adults, averaging between 55 and 60 years of age, with a male predominance (3:1 male-to-female ratio) [2, 9, 10]. Pediatric cases are rare, with common diagnostic delays owing to varied manifestations. ECD predominantly affects the skeletal system (95% of cases), cardiovascular disease, and the CNS. Retroperitoneal fibrosis is observed in approximately one-third of patients, and xanthelasma is a common skin feature [9, 11].

JXG, which is the most prevalent type of non-LCH histiocytosis, typically affects young boys and presents as a benign skin nodule that often resolves spontaneously. Extracutaneous or disseminated JXG, which may resemble ECD, requires a biopsy for accurate diagnosis. In cases of gain-of-function mutations in *BRAF*, *NRAS*, *KRAS*, or *MAP2K1*, the condition can be classified as ECD. Some experts view extracutaneous JXG and ECD as a continuum of the same disease [1, 2, 11, 12].

RDD is a rare condition with an estimated incidence of less than 10% of LCH, primarily affecting children and young adults, with a median age of onset of 20 years and minor male predominance [13]. It typically presents with massive bilateral cervical lymphadenopathy and constitutional symptoms, such as fever and weight loss. While primarily affecting the lymph nodes, extranodal involvement occurs in 43% of cases, frequently in the skin, soft tissue, upper respiratory tract, bones, eyes, and brain [11].

## Molecular pathophysiology

**Fig. 1:**
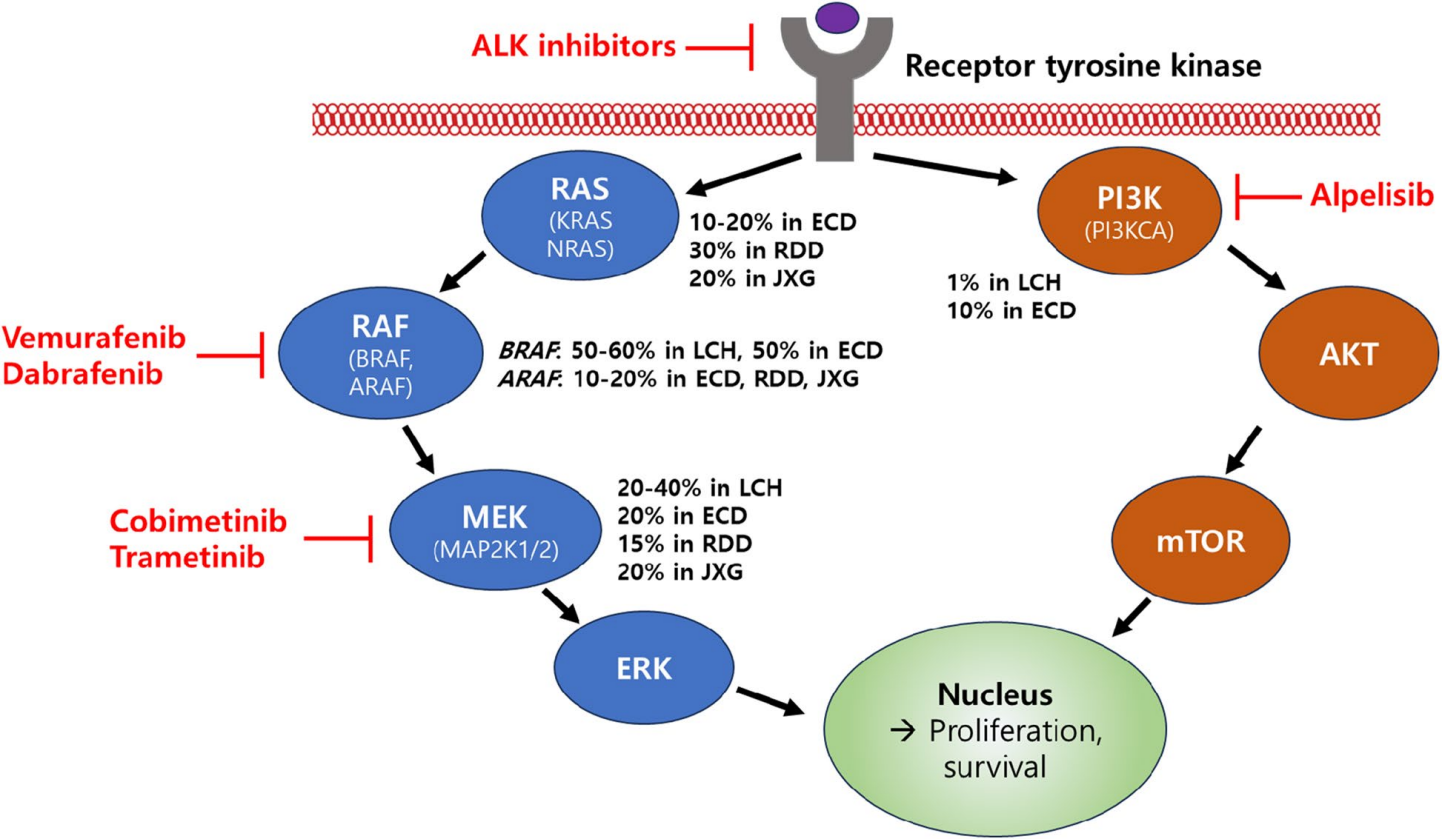
Genetic Alterations in Signaling Pathways and Targeted Inhibitors in Histiocytic Neoplasms. Abbreviations: LCH, Langerhans cell histiocytosis; ECD, Erdheim-Chester disease; RDD, Rosai-Dorfman disease; JXG, Juvenile xanthograunloma

Recent molecular studies have revealed that despite their distinct clinical and histological features, many histiocytoses share common molecular alterations. These primarily occur in the canonical MAPK and PI3K-AKT signaling pathways, indicating a degree of molecular similarity across different histological subtypes of the disease [2, 4, 6] (Table 2, Fig. 1).

**Table 2:** Frequency of genetic alterations in histiocytic neoplasms

**Genetic alterations**	**LCH**	**ECD**	**RDD**	**JXG**
*BRAF* alterations				
*BRAF* V600E	Frequent (50–60%)	Frequent (50%)	Rare	Rare^a^
*BRAF* deletions	Common (10%)	Rare	No	No
Other *BRAF* missense	Rare	No	No	No
*BRAF* fusions	Rare	Rare	No	Rare
*MAP2K1*	Common (20–40%)	Common (20–30%)	Common (15%)	Common (20–30%)
*RAS* isoforms (*KRAS*, *NRAS*)	Rare	Common (10–20%)	Common (30%)	Common (20–30%)
*ARAF*	Rare	Common (10–20%)	Common (10–20%)	Rare
*RAF1*	Rare	Rare	No	No
*PI3K* isoforms (*PI3KCA*, *PI3KCD*)	Rare	Common (10%)	No	Rare
*ALK* fusions	No	Rare	No	Rare^a^

*LCH* Langerhans cell histiocytosis, *ECD* Erdheim-Chester disease, *RDD* Rosai-Dorfman disease, *JXG* Juvenile xanthograunloma

^a^The *BRAF* V600E mutation and *ALK* fusion are exclusively observed in patients with systemic JXG and are not present in those with cutaneous JXG. These genetic alterations are relatively common in the systemic JXG cohort, with frequencies ranging from 20 to 30%

The most prevalent mutation is *BRAF* V600E [14, 15]. Other significant mutations include those in *MAP2K1* and a variety of rare gene mutations and fusions, such as those in *FAM73A*-*BRAF*, *MAP3K1*, and *ARAF* [6, 16, 17]. Non-Langerhans cell histiocytosis shares a high incidence similar to that of *BRAF* V600E mutations, along with genetic alterations in *ARAF*, *MAP2K1*, *PIK3CA*, *K/NRAS*, and gene fusions involving *BRAF*, *ALK*, *NTRK1*, and *ETV3*-*NCOA2* [6]. This highlights complex and diverse genetic landscapes.

The *BRAF* gene, a key player in the MEK-ERK signaling cascade in the MAPK pathway, is notably prevalent in histiocytic neoplasms. *BRAF* V600E mutations were found in 40–70% of LCH, 54% of ECD, 62.5% of HS, and 17% of systemic JXG patients. [15, 18–21]. Other *BRAF* mutations in LCH and histiocytic sarcomas include *BRAF* V600D, *BRAF* F595L, and *BRAF* V600insDLAT in LCH and histiocytic sarcoma [20, 22, 23]. These mutations are significant, as they indicate the clonal and neoplastic nature of these disorders due to ongoing MAPK signaling.

*MAP2K1* mutations, particularly prevalent in *BRAF* V600E-wildtype LCH (10–40% of patients), have been identified in 14% of ECD and 27% of *BRAF* V600E-wildtype JXG cases [6, 24, 25]. *MAP2K1* encodes an MEK1 kinase, which activates ERK1/2. These mutations predominantly occur in the N-terminal regulatory and catalytic domains of the gene, with some mutations activated, warranting further functional and treatment response evaluations [24, 25].

In addition, *ARAF* mutations have been discovered in 21%, 12.5%, and 18% of patients with ECD, RDD, and JXG, respectively, often occurring alongside *NRAS* mutations [25]. The frameshift mutations in *MAP3K1*, T799fs, and L1481fs in LCH suggest a potential loss-of-function [17, 22]. *RAS* isoform mutations, such as *NRAS* and *KRAS*, occur in 3–7% of ECD, 18% of JXG, 12.5–25% of RDD, and very rarely in LCH patients, while *HRAS* mutations are rare but are observed in HS patients with concurrent *BRAF* F595L mutation [11, 26–29]. In the PI3K-AKT pathway, *PIK3CA* mutations are present in 17% of *BRAF* V600E-wildtype ECD and 1.2% of LCH cases, with rare *PIK3CD* mutations in JXG [6, 27].

Several gene fusions have been reported to occur in histiocytic neoplasms. ALK-positive histiocytosis, a distinct entity first identified in 2008 in infants with liver and hematopoietic system involvement, is now known to have a broader clinical spectrum [30, 31]. Analysis of 39 cases revealed that it can present as a multisystemic disease in both infants and other patients, as well as a single-system disease [31]. This condition is characterized by frequent neurological involvement and is associated with *KIF5B-ALK* fusion, among other types. The study also noted various histological features and reported a significant response to ALK inhibition therapy, particularly in patients with neurological symptoms. Additionally, ALK fusions have been commonly identified in systemic JXG, noted in 8 of 15 patients, but are absent in cutaneous JXG cases. Moreover, non-LCH histiocytic neoplasms have been reported to exhibit gene fusion involving *BRAF* (such as *RNF11*-*BRAF* and *CLIP2*-*BRAF*) and *NTRK* (such as *LMNA*-*NTRK1*) [2, 25, 32].

## Treatment

### Overview

Histiocytic neoplasms display a wide range of clinical severity and treatment responses. Although some patients may not require treatment or can be treated with simple excision or topical therapy, systemic cases often require systemic chemotherapy. In LCH, the treatment approaches vary: observation or topical treatment for skin disease; curettage or curettage plus methylprednisolone injections for single bone lesions; and a 12-month systemic chemotherapy regimen of vinblastine and prednisone for multiple bone lesions or multisystem diseases, particularly in children and adolescents. Targeted therapy may be considered in relapsed or refractory conditions [4, 33].

JXG typically does not require treatment for a limited number of lesions, although excisional biopsy can be performed for cosmetic reasons. Systemic JXG is rare and lacks standardized treatment protocols. However, chemotherapy with vinca alkaloids and steroids should be considered. Clofarabine has been shown to be effective against systemic and CNS involvement in JXG [11].

ECD management varies with historical reliance on interferon therapy and close monitoring of side effects. However, recent advances in our understanding of the molecular pathogenesis have made targeted therapy a viable and effective option [11].

For RDD, treatment is often unnecessary except for the surgical management of large lymph nodes. However, in cases of multiorgan involvement or poor prognosis, steroids, chemotherapy, and MEK inhibitors, especially MAPK pathway gene mutations, are considered [11].

### Molecular target therapy

The discovery of *BRAF* and *MAP2K1* mutations has led to the development of targeted therapies that target the MAPK pathway (Fig. 1). First-generation BRAF inhibitors such as vemurafenib and dabrafenib, initially used for the treatment of metastatic melanoma, have shown efficacy in high-risk or refractory LCH cases. In particular, vemurafenib demonstrated significant responses in pediatric patients with refractory multisystem LCH, achieving a 100% overall response rate in an international study [34]. Dabrafenib, a selective BRAF inhibitor, reportedly has few adverse effects. A study in China with pediatric patients showed an overall objective response rate and a 75% disease control rate [35]. However, the high relapse rates following treatment interruption are a significant concern, indicating the need for further research to determine the optimal treatment duration and strategies for treatment withdrawal [34, 35].

Despite these promising results, the use of BRAF inhibitors is associated with challenges [36, 37]. These inhibitors are less effective against non-V600E *BRAF* mutations and can cause adverse effects, such as skin reactions, arthralgia, and the potential development of secondary cutaneous squamous cell carcinoma [8, 37–39]. Additionally, a significant issue is the rapid reactivation of LCH after discontinuation of these inhibitors [34, 35]. Current research focuses on combining BRAF inhibitors with MEK inhibitors to enhance their efficacy and reduce adverse reactions, a strategy that is being evaluated in ongoing clinical trials.

MEK inhibitors have shown efficacy against various histiocytic neoplasms and cobimetinib was recently approved by the FDA for adults with these conditions [37, 40]. However, the optimal treatment duration and impact of treatment interruption remain uncertain. A study including 22 adult patients with ECD, LCH, and RDD indicated progression in 17 of 22 cases after discontinuing therapy, although most patients regained a response upon treatment resumption, suggesting intermittent therapy as a potential approach [38]. This highlights the need for further research on treatment strategies and withdrawal studies for these complex diseases.

Additionally, evidence suggests that mutations in *PIK3CA* and other kinase genes such as *ALK*, *RET*, and *CSF1R* contribute to a subset of histiocytic disorders, with recent studies showing targeted therapies against these mutations. A recent case study reported the successful use of alpelisib, a PI3K inhibitor, in treating multisystemic LCH with *PIK3CA* mutation, achieving complete remission and validating *PIK3CA* as a novel therapeutic target [41].

## Conclusion

In conclusion, this study of histiocytic neoplasms revealed a complex and varied landscape of these diseases. Advances in molecular genetics have led to the identification of key mutations that drive these disorders, particularly those in the MAPK pathway, offering new avenues for targeted therapies. The efficacy of BRAF inhibitors, particularly in high-risk or refractory LCH cases, marks a significant advancement, despite challenges such as post-discontinuation relapse and adverse effects. Evolving therapeutic strategies, including the combination of *BRAF* and *MEK* inhibitors and the use of intermittent therapy, highlight the dynamic nature of treatment approaches. Additionally, targeted therapeutics, such as PI3K and ALK inhibitors, are likely to be beneficial in selected patient groups. The ongoing research and development of new treatment modalities underscores the need for further studies to optimize patient outcomes under diverse and challenging conditions.

## Data Availability

No datasets were generated or analysed during the current study.
